# Cutaneous and subcutaneous mass lesions in camels *(Camelus dromedarius)*: diagnostic features and treatment outcomes

**DOI:** 10.3389/fvets.2026.1709616

**Published:** 2026-04-24

**Authors:** El-Sayed El-Shafaey, Madeh Sadan, Naif Al-Gabri, Mohamed Hamed, Esam Mosbah, Gamal Karrouf, Fahad Abdullah Alshanbari

**Affiliations:** 1 Department of Surgery, Anaesthesiology and Radiology, Faculty of Veterinary Medicine, Mansoura University, Mansoura, Dakahlia, Egypt; 2 Department of Veterinary Surgery, Salam Veterinary Group, Buraydah, Qassim, Saudi Arabia; 3 Department of Clinical Sciences, College of Veterinary Medicine, Qassim University, Buraydah, Qassim, Saudi Arabia; 4 Veterinary Department, Faculty of Agriculture and Veterinary Medicine, Thamar University, Dhamar, Yemen; 5 Department of Pathology, Faculty of Veterinary Medicine, Mansoura University, Mansoura, Dakahlia, Egypt; 6 Department of Medical Biosciences, College of Veterinary Medicine, Qassim University, Buraydah, Saudi Arabia

**Keywords:** animals, camel, cutaneous, diagnostic imaging, mass, pathology

## Abstract

**Aim:**

Cutaneous and subcutaneous masses are frequently encountered in dromedary camels. However, comprehensive clinicopathological data regarding their prevalence, classification, and clinical outcomes remain limited. This study aimed to describe the diagnostic characteristics and surgical outcomes of skin masses in a hospital-presented camel cohort.

**Method:**

Between September 2023 and August 2024, a total of 213 dromedary camels were selected through purposive sampling based on the clinical presentation of various skin conditions across Riyadh, Al Qassim and Eastern provinces of Saudi Arabia. From this initial group, 92 cases of confirmed cutaneous or subcutaneous masses were included. Data on lesion type, anatomical location, age, sex, breed, and treatment outcome were collected and analyzed. Six types of skin masses were classified as neoplastic or non-neoplastic based on definitive histopathological biopsy features.

**Results:**

Non-neoplastic lesions were the most frequent (60.87%), with granulomas and chronic abscesses predominating. Neoplastic lesions accounted for 39.13% of cases, primarily consisting of fibromas (23.91%) and papillomas (10.87%). The most common anatomical sites for lesion occurrence were the hindlimbs (26.09%) and the head (17.39%). A higher frequency of lesions was observed in camels aged 5–10 years (75%) and in females (64.13%). Among breeds, Wadeh camels (56.52%) were most represented. Surgical excision resulted in complete primary recovery in 83.70% (77/92) of cases. While 16.30% (15/92) experienced postoperative complications, including edema, hemorrhage, infection, or recurrence, all were successfully managed.

**Conclusion:**

This study provides clinicians with valuable insights into the clinical frequency, diagnostic characteristics, and treatment outcomes of cutaneous and subcutaneous masses in dromedary camels. These findings establish a clinical baseline for classifying such lesions, supporting improved clinical decision-making and providing a foundation for future research in camelid medicine.

## Introduction

The dromedary camel is a socioeconomically important species, particularly for racing, milk, and meat production (1). Among the factors influencing the profitability and welfare of the camel industry, skin integrity is paramount (2, 3). Cutaneous and subcutaneous masses are frequently observed in clinical practice; their frequency often increases with age, presenting significant diagnostic and therapeutic challenges to veterinarians (4).

The etiology of these masses is multifactorial, including trauma, chronic infection, parasitic infestation, ultraviolet (UV) radiation, and congenital predispositions. Such lesions may arise from neoplastic processes or non-neoplastic conditions, such as chronic inflammatory lesions and cystic formations (1, 5). Regardless of their origin, these masses can cause significant discomfort and lead to systemic complications that diminish animal health and economic productivity, specifically affecting market value, body weight, milk yield, and fertility (6, 7). Consequently, characterizing the various types of skin masses is critical for determining a precise prognosis, selecting appropriate treatment modalities, and implementing effective herd management protocols.

A preliminary diagnosis is generally based on clinical examination, diagnostic imaging, and gross pathological evaluation (8). However, histopathological analysis remains the gold standard for definitive identification and classification (7, 9). While treatment options may include cryotherapy or chemotherapy, surgical excision remains the primary and most effective intervention (10, 11).

Despite the frequency of these masses in clinical settings, comprehensive studies addressing their classification, clinical presentation, and treatment outcomes in dromedary camels remain limited. Therefore, this study aimed to evaluate the clinical, ultrasonographic, and histopathological features, as well as the surgical outcomes, of cutaneous and subcutaneous masses within a hospital-based cohort of dromedary camels.

## Materials and methods

### Study animal

From September 2023 to August 2024, 213 dromedary camels presenting with various skin lesions that came from Riyadh, Al Qassim, and Eastern provinces which has a high density of camels, were examined at the University Veterinary Hospital, College of Veterinary Medicine, Qassim University, Saudi Arabia. Of these, 92 camels were included in the final study cohort based on the clinical presence of cutaneous or subcutaneous masses. The cohort comprised various breeds (52 Wadeh, 25 Sofor, and 15 Majaheem) and both sexes (33 males, 59 females), with an age range of 5–10 years (mean ± SD: 6.2 ± 2.5 years) and a weight range of 100–700 kg (mean ± SD: 300 ± 50 kg). The study protocol was approved by the Committee of Animal Welfare and Ethics at College of Veterinary Medicine, Qassim University, Saudi Arabia (QU-APC-2025).

### Inclusion and exclusion criteria

Camels were included if they presented with a clinically detectable cutaneous or subcutaneous mass, regardless of size, location, or duration. A complete clinical examination was performed, including a detailed physical, hematological, and biochemical examination. Definitive inclusion in the final analysis required a histopathological diagnosis provided by board-certified pathologists.

Camels were excluded if they exhibited systemic diseases, including active respiratory or gastrointestinal infections, metabolic disorders, or generalized dermatological conditions (e.g., dermatitis, pruritus or alopecia) not associated with a focal mass. These exclusions minimized systemic bias that could affect surgical recovery or the interpretation of localized lesion outcomes.

### Lesion classification

Masses were primarily classified by origin as neoplastic (epithelial vs. mesenchymal) or non-neoplastic (inflammatory or cystic) growth originating from the skin or subcutaneous tissues, including lesions located at muco-cutaneous junctions such as the vulva. Neoplastic lesions were graded according to the WHO Classification of Tumors. Non-neoplastic lesions were sub-classified as abscesses (purulent core with a fibrous capsule), granulomas (macrophage-rich with or without necrosis), or cysts (fluid-filled with an epithelial lining).

### Clinical examinations

Upon admission, all camels with cutaneous and subcutaneous masses underwent a thorough clinical examination of the lesion. The breed, age, and sex of each camel were recorded in conjunction with the type and location of each lesion. Physical examination of the vital signs and palpation of the superficial regional lymph nodes were conducted to detect enlargement suggestive of neoplastic metastasis.

### Ultrasonographic examinations

All animals with skin masses underwent ultrasonographic evaluation using 2.5–10 MHz transducers (SSD-500; Aloka, Tokyo, Japan), as per El-Shafaey et al. (2020). Camels were lightly sedated using intravenous (IV) injection of 0.2 mg/kg xylazine HCl (2%). For each case before imaging their internal architecture, the hair was clipped, the skin was cleaned with 70% isopropyl alcohol, and ultrasound acoustic coupling gel was applied to the skin surface. This preparation ensured high-quality imaging during the ultrasonographic examination of the cutaneous and subcutaneous masses. When needed, ultrasonography-guided fine-needle aspiration was performed to assist in the initial differential diagnosis. Images were subjectively interpreted with the clinical findings, and upon diagnosis of the type of lesion, the affected animal was treated according to the suitable treatment protocol.

### Surgical treatment

Before surgery, patients underwent hematological (complete blood count) and biochemical profiling (serum chemistry) to ensure the absence of anemia or organ failure. Following a 12-h fast, camels received preoperative cefquinome (1.5 mg/kg, IM) and meloxicam (0.5 mg/kg, IV). Deep sedation was achieved with xylazine (0.3 mg/kg, IV), and local anesthesia was provided via subcutaneous infiltration of 2% lidocaine (5 mg/kg) around the mass perimeter. After aseptic preparation of the surgical site, masses were excised using an elliptical skin incision and blunt dissection. Wounds were closed primarily with No. 2 polyglycolic acid; however, larger defects were managed via second-intention healing. Postoperative care of the treated camels included 4 weeks of stall rest and daily monitoring.

### Histopathological examinations

Tissue samples from the 92 confirmed cases were collected via biopsy for histopathological examination. For smaller, well-circumscribed masses, total excisional biopsy was performed, while deep incisional biopsies (using a 6–8 mm biopsy punch or scalpel) were obtained from larger or more invasive lesions. Excised tissues were fixed in 10% neutral buffered formalin for 48 h and processed routinely for paraffin embedding. The tissues were routinely processed with the automated processor (SLEE MTM, Germany) and embedded in paraffin wax, according to standard histological procedures. Five-micrometr sections were stained with hematoxylin and eosin (H&E), which were examined under a microscope equipped with a camera (Nikon ECLIPSE E200) for morphological evaluation. For cases of suspected squamous cell carcinoma (SCC), immunohistochemistry (IHC) for Cytokeratin (CK AE1/AE3) was performed at the pathology and cytology unit at King Fahd Specialist Hospital, Buraydah, to confirm epithelial origin and stromal invasion, distinguishing it from non-epithelial neoplasms like sarcomas or lymphomas.

### Statistical analysis

Data were analyzed using IBM SPSS Statistics (version 24.0). Categorical variables (breed, sex, age group, location, and outcome) were summarized as counts and percentages. Associations between lesion types and categorical variables were assessed using the Chi-squared (χ^2^) test of independence. Normality was confirmed via the Shapiro–Wilk test, and mean ages across lesion types were compared using ANOVA. Statistical significance was set at a *p*-value less than 0.05.

## Results

### Mass lesion frequency, distribution, and clinical associations

The demographic profile of the study cohort (*n* = 213 camels) and the associated frequency of mass lesions are detailed in Table 1. The Wadeh breed was the most represented (44.13%) and demonstrated a significantly higher proportion of lesions (56.52%) compared to other breeds (*p* < 0.001). Although females comprised the majority of the cohort (60.56%), they exhibited a significantly higher lesion frequency (64.13%) than males (35.48%; *p* = 0.01). Furthermore, camels aged 5 to 10 years represented the largest portion of the study population (60.56%) and accounted for the highest frequency of identified lesions (75.00%; *p* < 0.001). Anatomically, the hindlimbs (26.09%) and head (17.39%) were the most common sites for all mass types.

**Table 1:** Breed, sex, and age distribution of camel^,^ cohort and associated incidence and odds of cutaneous and subcutaneous mass lesions.

Subject	Category	Total examined (*n* = 213)	Affected (*n* = 92)	Incidence (%)	*p*-value
Breed	Wadeh	94 (44.13%)	52	56.52%	<0.001
	Sofor	58 (27.23%)	25	27.17%	
	Majaheem	61 (28.64%)	15	16.31%	
Sex	Male	84 (39.44%)	33	35.87%	
	Female	129 (60.56%)	59	64.13%	0.01
Age	< 5 years	49 (23.01%)	12	13.04%	
	5–10 years	129 (60.56%)	69	75.00%	<0.001
	> 10 years	35 (16.43%)	11	11.96%	

*p-*values were derived from Chi-squared tests comparing the incidence rates between categories for each characteristic.

**Table 2:** Frequency rates (%) of different cutaneous and subcutaneous mass lesions for camel^,^ cohort.

Lesion (*n* = 92)	Type	Subtype	Number		Frequency	Total *p*-value
Neoplastic (*n* = 36)	Fibroma	Hard	16	20	21.74%	(*n* = 36; Prevalence = 39.13%)
		Soft	4			
	Papilloma	Fibro	6	10	10.87%	
		Myxo	1			
		Viral	3			
	Carcinoma	Squamous cell	5	6	6.52%	
		Adeno	1			
Non-neoplastic (*n* = 56)	Chronic inflammatory masses	Granuloma	22	48	52.17%	(*n* = 56; Prevalence = 60.87%)
		Chronic abscesses	10			
		Bursitis	6			
		Caseous lymphadenitis	5			
		Chronic lymphangitis	5			
	Chronic cystic lesions	Dermoid	8	8	8.70%	

### Mass lesion spectrum and relative frequency

Of the 92 camels presenting with cutaneous or subcutaneous masses, non-neoplastic lesions were the most frequent, constituting 60.87% of cases, a significantly higher proportion (*p* < 0.049) than neoplastic lesions (39.13%).

**Figure 1:**
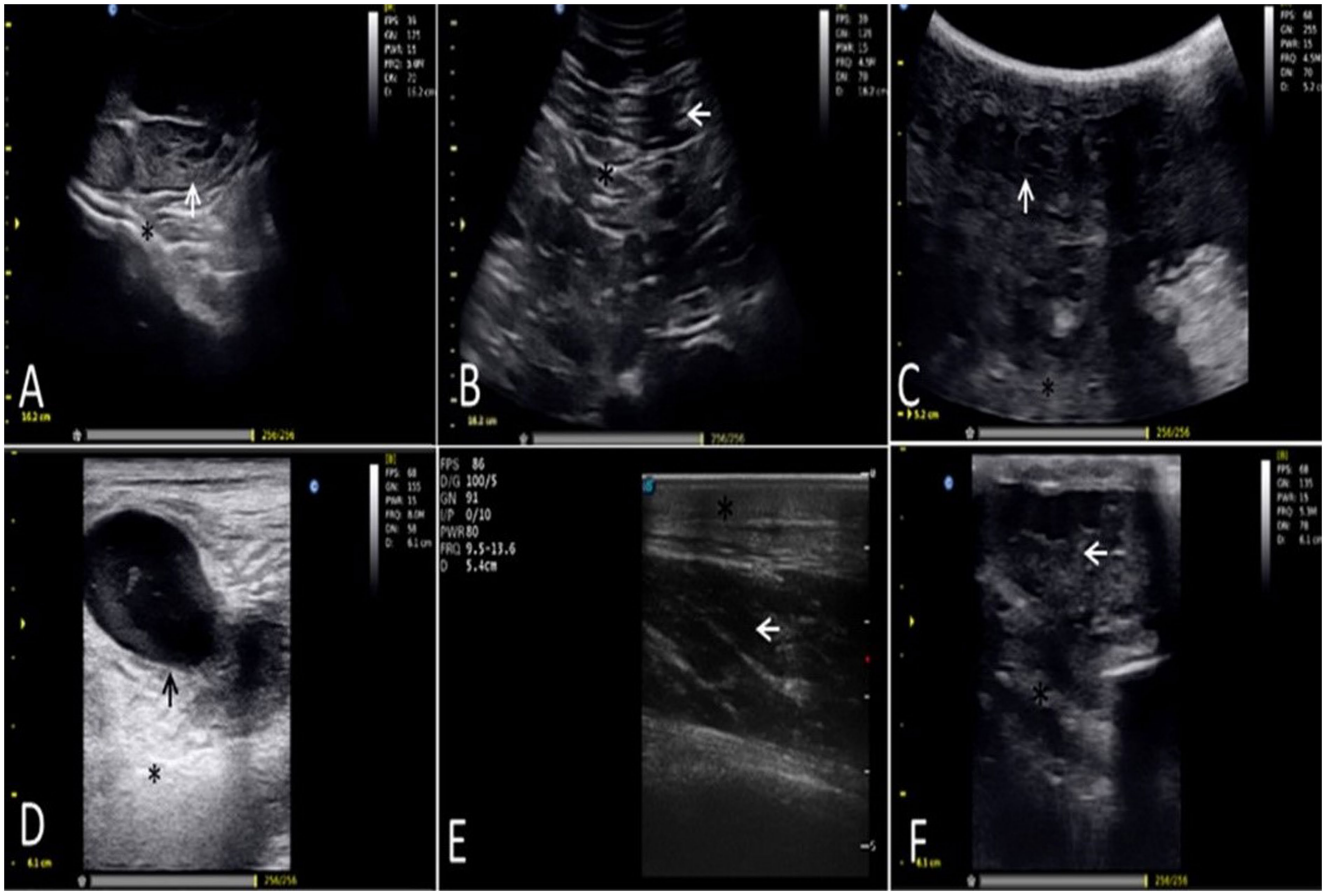
Ultrasound images correspond to the most prevalent cutaneous and subcutaneous mass lesions in dromedary camels. **(A)** A neoplastic mass (fibroma), showing a distinct, isoechoic to hyperechoic structure (white arrow) with posterior acoustic enhancement (asterisk). **(B)** A chronic abscess, demonstrating heterogeneous hypoechoic to anechoic content (white arrow) with internal septations (asterisk). **(C)** Serofibrinous bursitis, featuring a thick echogenic capsule (asterisk) surrounding a fibrous mass with minimal fluid (white arrow). **(D)** Caseous lymphadenitis, showing a characteristic hypoechoic cystic area (black arrow) within an echogenic lymph node (asterisk). **(E)** Chronic lymphangitis, with an echogenic wall (asterisk) and mixed internal echogenicity (white arrow). **(F)** A dermoid/epidermoid cyst, displaying mixed echogenicity (white arrow) and echoic foci suggestive of keratin or hair (asterisk).

**Figure 2:**
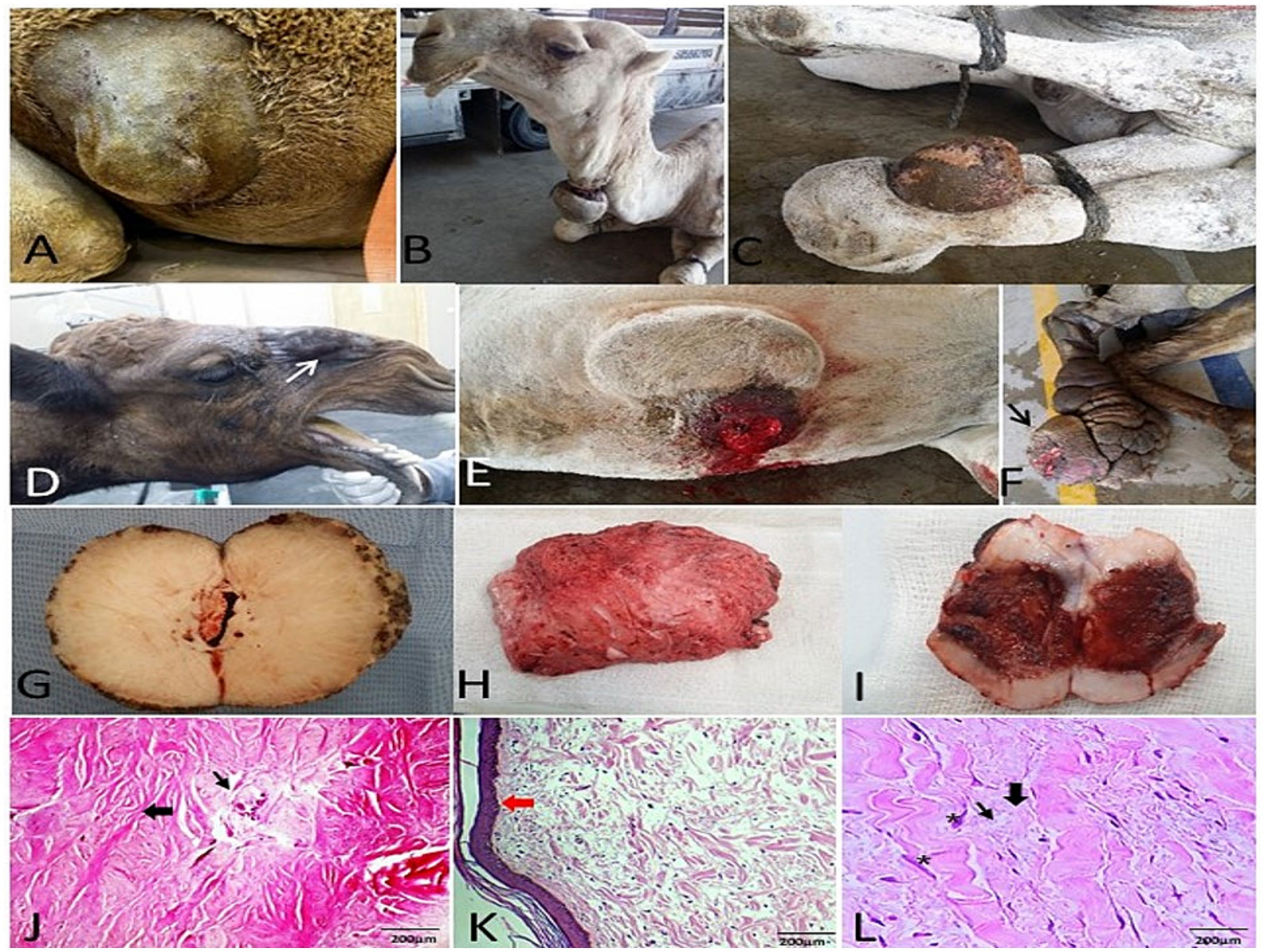
Gross and histopathological features of fibromas in camels. Gross morphology of **(A–C)** hard and **(D–F)** soft fibromas. **(G–I)** Cut surfaces reveal firm, gray-white tissue, sometimes congested. **(J,K)** Histomicrograph of a hard fibroma: Thin epidermis (red thick arrows) with the underlying dermis characterized by dense, haphazardly arranged collagen bundles interspersed with fibroblasts (black thick arrows) nearest blood vessels (thin arrows) (H&E stain; scale bar: 200 μm). **(L)** Histomicrograph of a soft fibroma: Swirling bundles of stellate fibroblasts (asterisks), scant collagen fibers (thick arrow), and prominent vasculature (thin arrow) (H&E stain; scale bar: 200 μm).

**Figure 3:**
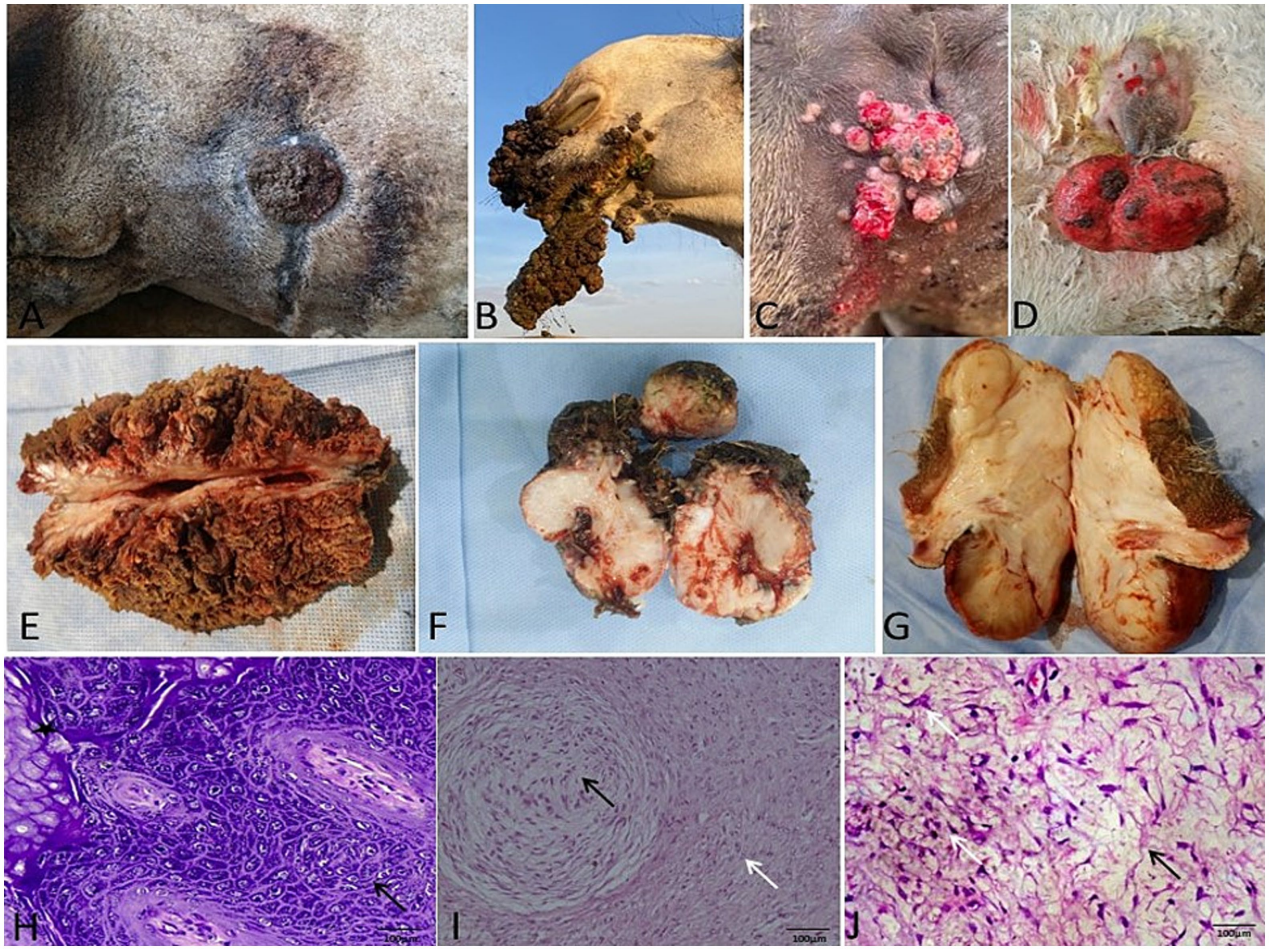
Gross and histopathological features of papillomas in camels. Gross morphology of **(A)** fibropapilloma, **(B,C)** viral papilloma, and **(D)** myxopapilloma. **(E–G)** Corresponding cut sections: fibropapilloma (gray-white, firm), viral papilloma (cauliflower-like), and myxopapilloma (yellowish, blood-tinged). **(H)** Histomicrograph of viral papilloma: papilliferous projections (black arrow) exhibiting marked hyperkeratosis and acanthosis (star) (H&E). **(I,J)** Histomicrograph of myxopapilloma: at low and high magnification, characterized by spindled cells (white arrow) within an abundant myxoid stroma (black arrow) (H&E; 100 μm).

**Figure 4:**
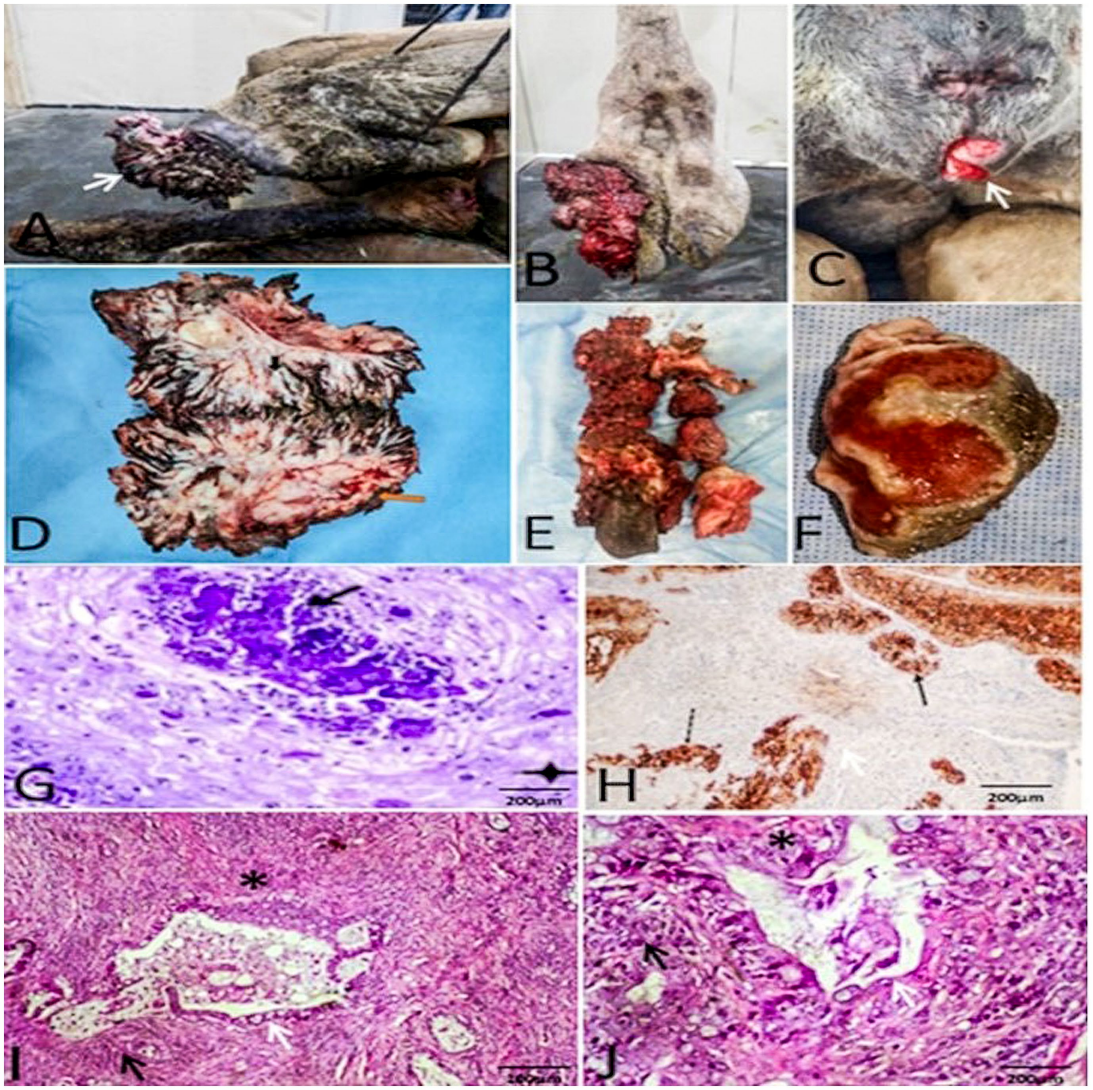
Gross and histopathological features of carcinomas in camels. Gross morphology of **(A,B)** squamous cell carcinoma in camels (SCC) and **(C)** vulvar adenocarcinoma in a female Wadeh camel. **(D–F)** Cut sections: SCC shows ulcerated proliferations; adenocarcinoma is firm and necrotic. **(G)** Histomicrograph of SCC: Nest of neoplastic cells (star) and keratin pearls (thick arrow) (H&E; 200 μm). **(H)** Immunohistochemistry: Diffuse cytokeratin positivity confirms epithelial origin (arrows). **(I,J)** Histomicrograph of adenocarcinoma: Malignant glandular acini (white arrows) lined by atypical epithelium (black arrow) within a desmoplastic stroma (asterisk) (H&E; 200 μm).

**Figure 5:**
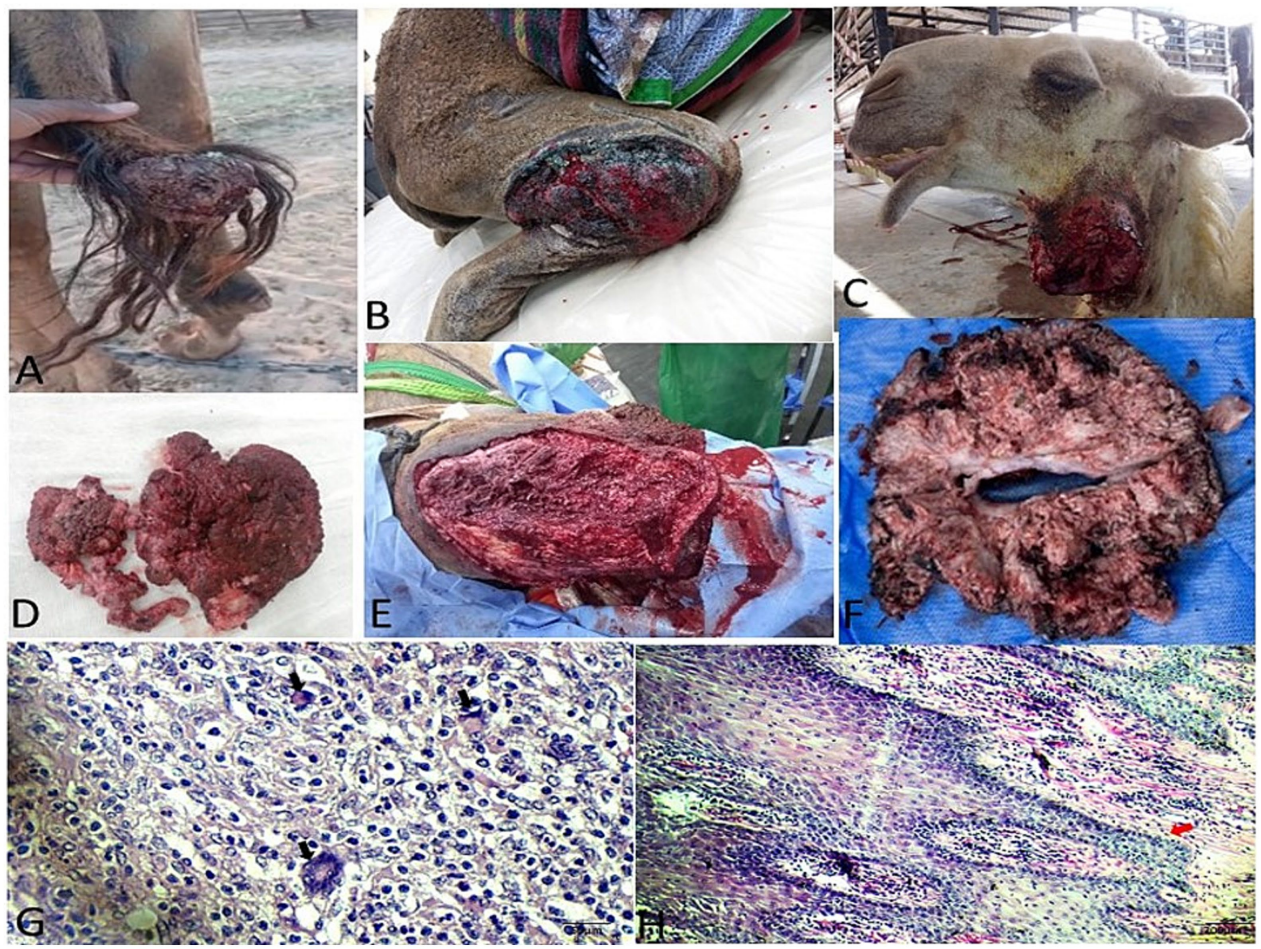
Gross and histopathological features of granuloma in camels. Gross morphology of **(A,B)** fibrogranuloma and **(C)** pyogranuloma. **(D–F)** Cut surfaces show fleshy, ulcerative tissue with suppuration in the pyogranuloma. **(G,H)** Histomicrograph of fibro-granuloma: marked epidermal hyperplasia, prominent rete ridges (red arrows) followed with a nodular to diffuse inflammatory infiltration composed of mixture neutrophils, macrophages, and giant cells (black arrows) (H&E; 100, 200 μm).

**Figure 6:**
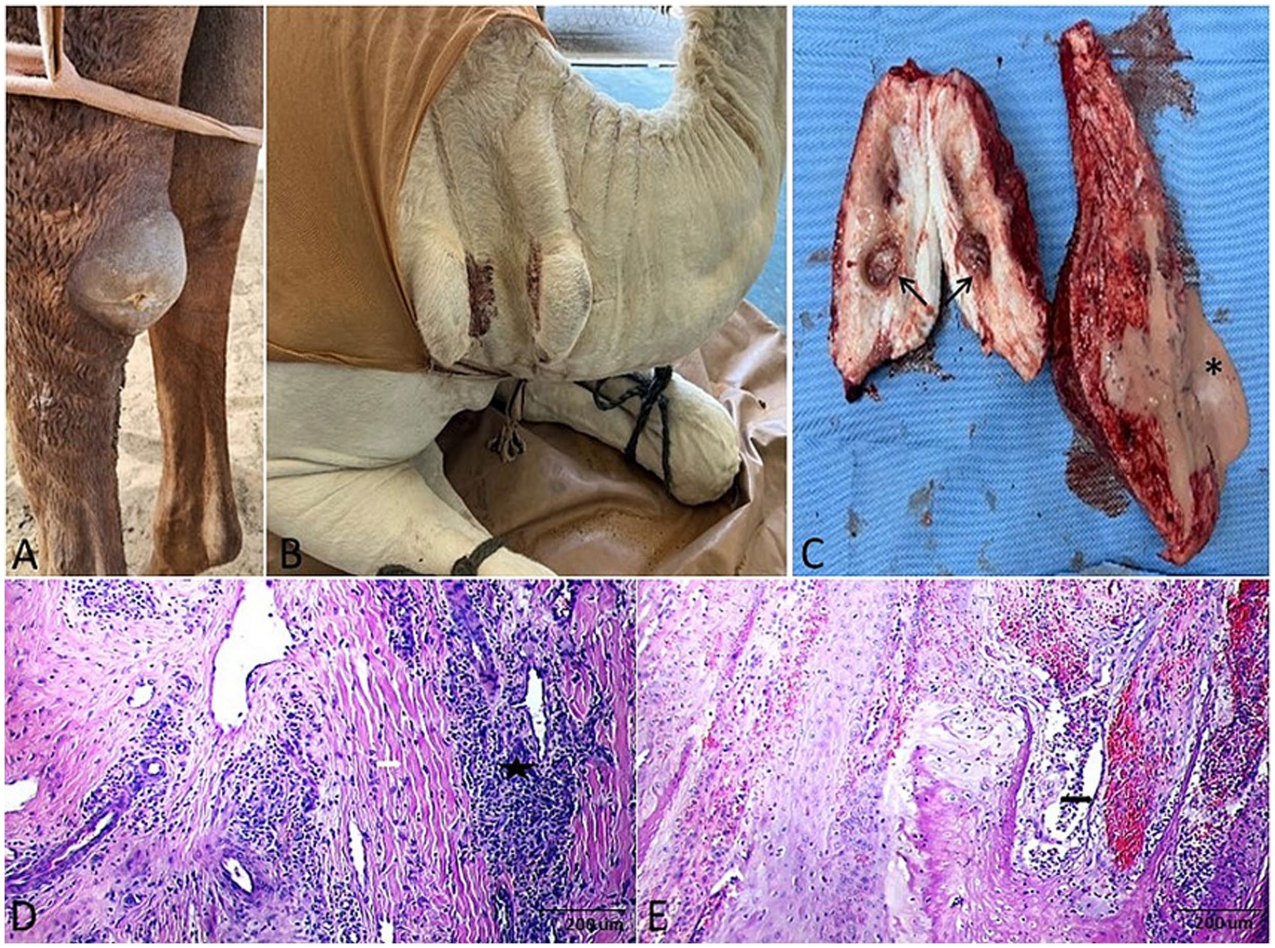
**(A,B)** Gross morphology of chronic abscess in camels. **(C)** Cut section reveals a thick fibrous wall, suppurative tracts (arrows), and a necrotic core (asterisk). **(D,E)** Histomicrograph: Thick dense connective tissue (white arrow), individual aggregates of chronic granulomatous inflammation (star), necrosis, and sometimes replaced by hemorrhages (black arrow) (H&E; 200 m).

**Figure 7:**
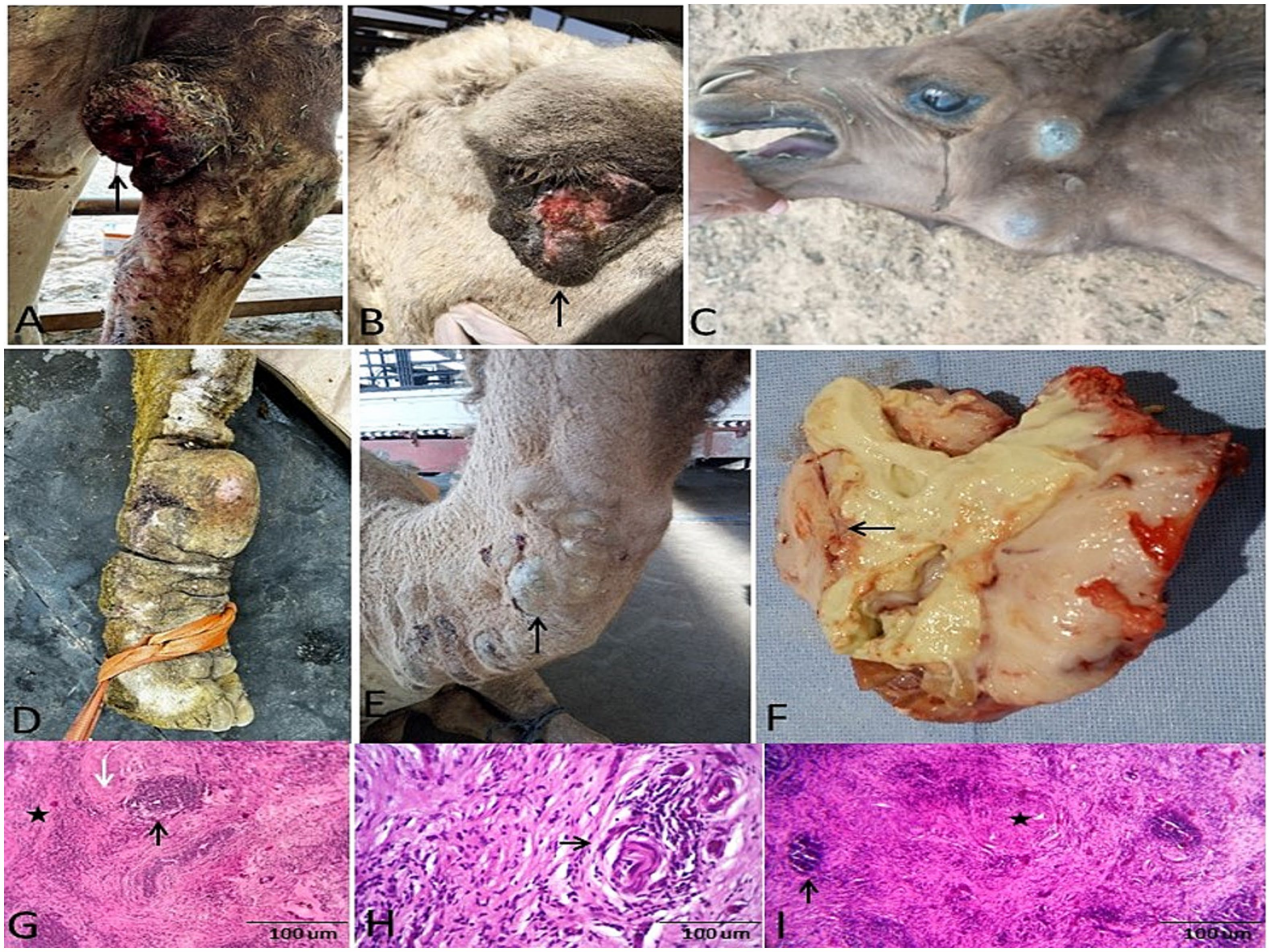
Spectrum of chronic inflammatory conditions: bursitis, dermatitis, lymphadenitis, and lymphangitis. Gross morphology of **(A)** olecranon bursitis, **(B)** granulomatous dermatitis, **(C)** caseous lymphadenitis, and **(D,E)** chronic lymphangitis. **(F)** Cut lymph node with a caseous necrotic focus (arrow). **(G)** Histomicrograph of bursitis: dense connective tissue (white arrow), capillaries (star), and inflammatory cell aggregations (black arrow) (H&E; 100 μm). **(H,I)** Histomicrograph of caseous lymphadenitis: encapsulated pyogranulomatous reaction characterized by an infiltration of chronic inflammatory cells (black arrow) surrounding the many small blood vessels, with extravasated erythrocytes (star).

**Figure 8:**
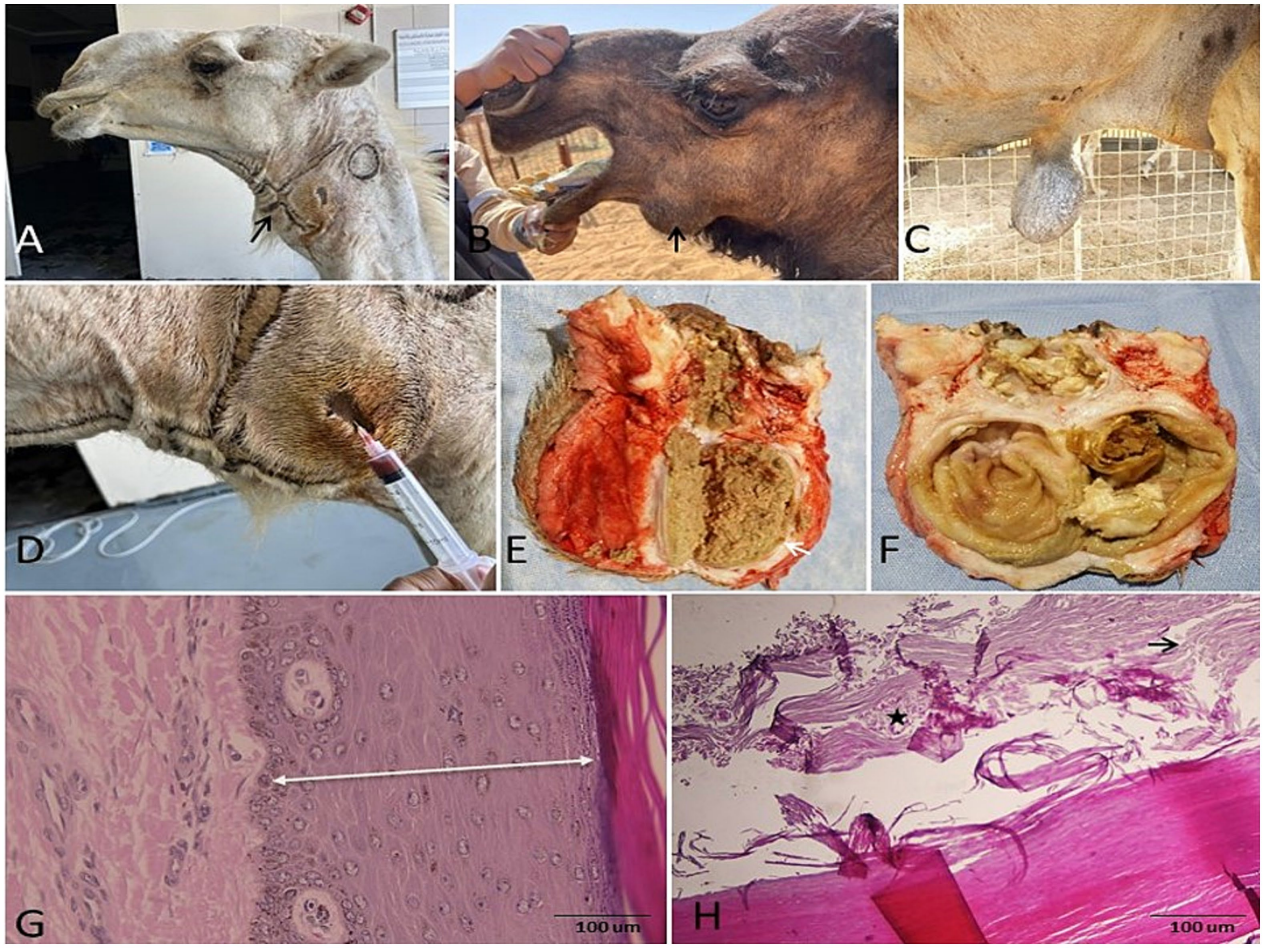
Dermoid and epidermoid cysts in camels. Gross morphology of dermoid cysts on the **(A,B)** neck/submandibular area and **(C)** a large umbilical epidermoid cyst. **(D)** Aspirated black, watery fluid from a dermoid cyst. **(E,F)** Cut sections of an epidermoid cyst showing sandy, doughy lamellar contents. **(G)** Histomicrograph of a dermoid cyst: cystic cavity which lined by a keratinizing epidermal stratified squamous epithelium (duple-head white arrow) including all dermal layers as well as granular epithelium (H&E; 100 μm). **(H)** Histomicrograph of an epidermoid cyst: lumen filled with desquamated keratin, often appearing as laminated, concentric, ghost cell (shadow cell) morphology (star), and waxy keratin scales (black arrow) with absence of dermal adnexa (H&E; 100 μm).

Lesions were categorized by histopathological diagnosis, with the relative frequency of specific entities detailed in Figures 1–8 and Tables 1–4. The most common neoplasms were fibromas (21.74%) and papillomas (10.87%). Among non-neoplastic masses, granulomas (23.91%) and chronic abscesses (10.87%) predominated. Granulomas were further subtyped into fibrogranuloma and pyogranulomas.

**Table 3:** Frequency rates (%) of different cutaneous and subcutaneous mass lesions by location for camel^,^ cohort.

Lesion	Type	Location									
		Head	Neck	Thorax	Abdomen	Forelimb	Hindlimb	Prepuce	Udder	Vulva	Tail
Neoplastic (*n* = 36)	Fibroma (*n* = 20)	1	2	3	5	2	6	0	0	0	1
	Papilloma (*n* = 10)	2	0	0	2	0	2	1	0	1	2
	Carcinoma (*n* = 6)	0	0	0	1	3	1	0	0	1	0
Non-neoplastic (*n* = 56)	Chronic inflammatory masses (*n* = 17)	11	7	5	2	5	15	1	1	0	1
	Chronic cystic lesions (*n* = 8)	2	2	0	4	0	0	0	0	0	0
Total	92	16	11	8	14	10	24	2	1	2	4
Frequency %		17.39%	11.96%	8.69%	15.22%	10.87%	26.09%	2.17%	1.09%	2.17%	4.35%

**Table 4:** Frequency rates (%) of different cutaneous and subcutaneous mass lesions by age and sex in the camel^,^ cohort.

Lesion	Type	Age (Year)			Sex		Breed			Treatment outcomes	
		< 5	5–10	> 10	Male	Female	Wadeh	Sofor	Majaheem	R	RC
Neoplastic (*n* = 36)	Fibroma (*n* = 20)	2	15	3	8	12	10	5	5	18	2
	Papilloma (*n* = 10)	2	7	1	3	7	7	2	1	9	1
	Carcinoma (*n* = 6)	1	4	1	1	5	6	0	0	4	2
Non-neoplastic (*n* = 56)	Chronic inflammatory masses (*n* = 17)	5	39	4	16	32	26	17	5	39	9
	Chronic cystic lesions (*n* = 8)	2	4	2	5	3	3	1	4	7	1
Total	92	12	69	11	33	59	52	25	15	77	15
Frequency %		13.04%	75.00%	11.96%	35.87%	64.13%	56.52%	27.17%	16.31%	83.70%	16.30%

R, Recovered. RC, Recovered with complications of edema, hemorrhage, infection, or recurrence.

### Diagnostic correlation

#### Ultrasonographic findings

Ultrasonography consistently correlated with definitive histopathology. Neoplasms typically appeared as well-defined, isoechoic to hyperechoic masses with posterior acoustic enhancement (Figures 1A,D,E). Skin masses of abscesses and affected lymphatic structures revealed heterogeneous, hypoechoic to anechoic cavities with internal septa and thick, echogenic capsules (Figure 1B), features highly suggestive of caseous lymphadenitis or chronic lymphangitis. Cystic lesions, such as bursitis and dermoid cysts, displayed anechoic fluid encapsulated by well-demarcated echogenic walls creating posterior acoustic enhancement; internal echoes or hair fragments helped differentiate specific cystic types (Figures 1C,F).

#### Histopathological findings

Histopathology provided definitive classification for all excised cutaneous and subcutaneous masses. Fibromas were divided into “hard” subtypes (dense collagen, scant fibroblasts; Figures 2J,K) and “soft” subtypes (loose collagen, higher fibroblast density; Figure 2L). Three papilloma subtypes were identified: viral (papillary proliferation; Figure 3G), fibro- (fibroblastic stroma; Figure 3H), and myxopapilloma (myxoid matrix; Figure 3I) subtypes.

Squamous cell carcinoma (SCC) was identified by invasive keratinizing islands and confirmed via positive pan-cytokeratin (CK AE1/AE3) immunostaining (Figures 4A–J). Vulvar adenocarcinoma presented as malignant glandular structures with a marked host response, including a desmoplastic stroma, a dense inflammatory infiltrate, and extensive areas of necrosis and suppuration (Figures 4I,J).

Non-neoplastic fibrogranulomas consisted of a dermal nodules of granulation with significant collagen and mixed inflammation (Figures 5G,H), while pyogranulomas featured central necrotic cores and microabscess formation (Figure 5I). Chronic abscesses exhibited necrotic debris and neutrophils surrounded by dense fibrous walls and mixed inflammatory cells (Figures 6D–F). Chronic bursitis was subclassified as cystic(elbow joint), serofibrinous (elbow joint), or fibrous (carpal and stifle joint) based on the degree of fibrosis and fluid exudation. Microscopically, they showed a synovial-like membrane covering a fibrous capsule with evidence of chronic inflammation (vessel congestion, perivascular mononuclear cells) (Figure 7G). Lymphatic involvement presented as suppurative lymphadenitis or lymphangitis with liquefactive necrosis, pus formation, and lymphoid depletion (Figures 7H,I). Finally, dermoid and epidermoid cysts were characterized by keratinized stratified squamous epithelial linings containing lamellar keratin (Figure 8G). The wall of dense collagenous tissue without adnexal structures (Figure 8H) differentiated these from other cystic structures and explained their well-demarcated, anechoic sonographic appearance.

### Treatment outcomes

Surgical excision was the primary and highly effective treatment. Of the 92 treated camels, 77 (83.70%) recovered without incident. Postoperative complications occurred in 15 cases (16.30%), including edema, hemorrhage, infection, and recurrence (2 SCCs, 1 viral papilloma, and 2 pyogranulomas), all of which were successfully managed during the six-month follow-up period.

## Discussion

Cutaneous and subcutaneous masses in camels present a diagnostic challenge due to limited specialized research. While many such masses are non-neoplastic, including abscesses, granulomas, and cysts, their presence, especially alongside lymphadenopathy, necessitates thorough investigation to rule out neoplasia or immune-mediated disorders (9, 11–13). This study provides a comprehensive evaluation of the clinical frequency, diagnosis, and treatment of these masses in a cohort of 92 dromedary camels. We identified six types of neoplastic and tumor-like lesions (granuloma, abscess or cyst), with non-neoplastic cases occurring more frequently (60.87%, *n* = 56/92) than neoplastic ones (39.13%, *n* = 36/92). Granulomas and chronic abscesses were the most common non-neoplastic findings, while fibromas and papillomas were the most frequent neoplasms.

The distribution of these lesions appears influenced by a combination of biological, environmental, and management-related factors (6, 14). In domestic animals, prolonged exposure to ultraviolet (UV) radiation is a recognized risk factor for cutaneous neoplasms, such as squamous cell carcinoma (5, 6). Given that camels in Saudi Arabia experience intense daily sunlight, cumulative UV exposure may contribute to lesion development (13, 14). In addition to solar radiation, other factors—including hormonal influences, chronic inflammation, traumatic injuries, and environmental stressors—contribute to the complex etiology of skin tumors across different species. Additionally, the high frequency of fibromas and granulomas observed here suggests a potential link to chronic trauma and irritation (14). Repetitive micro-trauma can induce fibroblastic proliferation or chronic inflammation, as evidenced by our histopathological findings of fibrogranulomas. Furthermore, the presence of papillomas aligns with the known role of papillomaviruses in camelids (5, 13). Variations in viral, fibro-, and myxopapillomas may reflect differing viral strains or host immune responses, providing a basis for future genetic and viral research. Implementing preventive measures, such as improved bedding and vector control, might help reduce the frequency of trauma-related lesions.

Breed characteristics may also play a role in lesion susceptibility (15, 16). In this cohort, Wadeh camels showed the highest frequency of skin lesions (56.52%), among the various camel breeds. While this could suggest a genetic predisposition or a higher sensitivity of white-coated breeds to UV radiation, it may also simply reflect local population demographics where Wadeh camels are more numerous. Further genetic studies are required to clarify these associations.

Regarding age, lesions were predominantly observed in camels aged 5–10 years (75.00%). This suggests that adult camels may be more prone to skin masses, potentially due to increased occupational exposure, recurrent trauma, and cumulative solar radiation (10, 14, 17). Similarly, a higher proportion of lesions was recorded in females (64.13%). This may be linked to management practices, as females are typically kept longer for breeding and milk production, whereas males are often slaughtered younger for meat (2, 3). It is also possible that physiological stressors related to pregnancy and lactation could influence susceptibility, though this requires further investigation (18).

Anatomically, lesions were most frequent on the hind limbs (26.09%) and head (17.39%). The hind limbs are subject to significant weight-bearing stress and trauma, while the head is a high-risk site for actinic damage and solar-induced carcinogenesis (1, 19). These findings underscore the importance of a systematic diagnostic approach that considers the animal’s history and environmental risks.

Diagnostic imaging and pathology remain essential for accurate characterization. Ultrasonography proved to be a valuable, non-invasive tool for distinguishing between neoplastic (isoechoic/hyperechoic) and non-neoplastic (hypoechoic/anechoic) architectures, helping guide surgical versus conservative management (8, 11, 20–22). However, histopathology remains the gold standard for definitive diagnosis. In this study, non-neoplastic masses typically exhibited mixed inflammatory cell infiltrates, necrosis, and fibrosis. For complex cases, such as invasive SCC, the selective use of pan-cytokeratin immunohistochemistry (CK AE1/AE3) was crucial (7, 15). By confirming the epithelial origin and highlighting stromal invasion, IHC resolved diagnostic challenges where standard H&E staining was insufficient, particularly in distinguishing poorly differentiated SCC from cytokeratin-negative sarcomas (16, 23).

Histopathological differentiation is critical for establishing a prognosis. For instance, fibromas must be carefully distinguished from low-grade fibrosarcomas; our diagnoses relied on the absence of significant cellular atypia, mitotic figures, and infiltrative growth patterns (20, 24, 25). Similarly, the rare myxopapilloma identified in this study, characterized by stellate cells in a myxoid matrix, was differentiated from malignant myxoid tumors, such as myxosarcomas, based on cell uniformity and the lack of malignancy indicators, consistent with previous reports (6, 26).

Early and accurate diagnosis remains a significant challenge, as lesions often progress extensively before clinical intervention and can easily be misidentified (1, 14, 27–29). The integration of clinical examination, diagnostic imaging, and histopathology is vital for refining differential diagnoses, expanding viable treatment options, and potentially improving the prognosis for such skin lesions in camels.

Prompt intervention is a key factor in improving surgical outcomes (30). If left untreated, masses may enlarge significantly, causing secondary damage to adjacent structures. While various treatment options exist, surgical excision remains the primary and most effective approach (10, 31). In this study, all cases were treated surgically, resulting in a high recovery rate (83.70%). Postoperative complications occurred in 16.30% of cases, including edema, hemorrhage, infection, and recurrence, but were managed successfully. These findings suggest that complications are more likely when dealing with large masses or those located in high-tension areas or near vital structures.

This study has certain limitations that warrant acknowledgment. First, we did not evaluate all potential risk factors or specific management practices associated with these lesions. Second, the molecular etiopathogenesis of the neoplastic lesions was not explored. Finally, while immunohistochemistry (IHC) was used for specific cases, a broader panel of markers for comprehensive differential diagnosis was not employed. Future research involving larger populations across diverse geographical regions is recommended to investigate genetic mutations, abnormal signaling pathways, and viral inclusion bodies. Additionally, exploring advanced IHC markers and non-surgical treatment options for metastatic disease is necessary to advance camel oncology.

## Conclusion

This study provides clinicians with valuable insights into the frequency, diagnostic features, and treatment outcomes of cutaneous and subcutaneous masses in dromedary camels. Precise early diagnosis followed by prompt surgical intervention generally results in favorable outcomes, preserving the health and productivity of the animals. Furthermore, this work establishes a clinical baseline for the classification of these lesions, providing a foundation for improved clinical decision-making and future research into the genetic and viral origins of camelid skin masses.

## Data Availability

Data and materials are provided within the manuscript or supplementary information files.
